# Defects, diffusion and dopants in Li_8_SnO_6_

**DOI:** 10.1016/j.heliyon.2021.e07460

**Published:** 2021-07-02

**Authors:** Navaratnarajah Kuganathan, Andrei L. Solovjov, Ruslan V. Vovk, Alexander Chroneos

**Affiliations:** a Department of Materials, Imperial College London, London, SW7 2AZ, United Kingdom; b Faculty of Engineering, Environment and Computing, Coventry University, Priory Street, Coventry, CV1 5FB, United Kingdom; c B. Verkin Institute for Low Temperature Physics and Engineering, NAS of Ukraine, 47 Nauky Avenue, Kharkiv, 61103, Ukraine; d V. Karazin Kharkiv National University, 4 Svobody Square, Kharkiv, 61077, Ukraine

**Keywords:** Li_8_SnO_6_, Defects, Diffusion, Dopants, Atomistic simulation

## Abstract

Octalithium tin (IV) oxide (Li_8_SnO_6_) is an important electrode material considered for lithium ion batteries (LIBs) because of its high lithium content. We employed atomistic simulations to examine the intrinsic defects, diffusion of Li-ions together with their migration energies and solution of potential dopants in Li_8_SnO_6_. The most thermodynamically favourable intrinsic defect is the Li Frenkel which increases the concentration of Li vacancies needed for the vacancy mediated diffusion of Li-ions in Li_8_SnO_6_. The calculated activation energy of migration of Li-ions (0.21eV) shows that the Li-ion conductivity in this material can be very fast. Promising isovalent dopants on the Li and Sn sites are Na and Ti, respectively. Doping of Ga on the Sn site can facilitate the formation of Li interstitials as well as oxygen vacancies in Li_8_SnO_6_. While the concentration of Li interstitials can enhance the capacity of this material, oxygen vacancies together with Li interstitials can lead to the loss of Li_2_O in Li_8_SnO_6_.

## 1 Introduction

LIBs have the potential to improve energy efficiency and reduce the toxic pollutants releasing from burning fosile fuels [1, 2, 3, 4, 5]. The properties of the electrode or electrolyte material have a major impact on battery performance. Considerable research acticvity has been performed to identify suitable electrode materials, both experimentally and theoretically [6, 7, 8, 9, 10]. Recently, cobalt nickel sulfide nanoneedle arrays [11], titanium nitride nanoparticles-sulphur composites [12] and two dimensional hexagonal CoMoO_4_ nanosheets have been considered as electrode materials [13]. Nanoporous electrode materials have also been recently synthesized to enhance the electrochemical performance [14, 15]. The search for novel electrode materials is still relevant in order to produce materials that are low cost, high abundance and non-toxic.

Sn-based oxides are promising materials in the development anode for LIBs due to their high capacity together with electrochemical performance arising from the reduction of capacity fading in comparison with pure tin [16, 17, 18, 19, 20, 21]. Previous experimental and theoretical studies examined the capacity, cycling stability and electrochemical performance and defect properties including diffusion and dopants in Li_2_SnO_3_ [22, 23, 24]. “Li-rich” Li_8_SnO_6_ is another Sn-based oxide material has attracted considerable attention for its use as an electrode material owing to its high Li-ion content; leading to the release of more than one lithium per formula unit theoretically [25]. However, the experimental extraction of the exact number of Li-ions is not available. The loss of Li_2_O can be facilitated by the introduction of lithium and oxygen vacancies in the lattice. The main drawback of forming Li_2_O is capacity loss together with reduction in Coulombic efficiency [26]. Luo *et al.* [27], performed density functional theory (DFT) simulations to show that the oxygen redox yields a high voltage plateau of over 4.0 V (vs Li/Li^+^). Furthermore, Li-ion diffuses fast in this material with an activation energy of 0.43 eV. In the literature, there are no more experiemental or theoretical studies available on the electrochemical performance, defects, diffusion pathways and dopants properties in Li_8_SnO_6_. Computer modelling techniques can be used to understand the fundamental properties of Li_8_SnO_6_ and optimise its performance via an appropriate doping mechanism. In previous studies, a variety of materials including energy materials have been modelled using classical and DFT simulations [28, 29, 30, 31, 32, 33]. The discovery of novel electrode or electrolyte materials together with better understanding of electrochemical properties is a main progress from the computational modelling. However, there is a great challenge to model solid electrolyte-electrode interfaces and disordered or amorphous phases of molecular materials [34].

In this study, simulations based on pair-wise potentials are used to study of intrinsic defects, Li-ion diffusion pathways and doping of isovalent cations [Na^+^, K^+^ and Rb^+^ on the Li site and Si^4+^, Ge^4+^, Ti^4+^, Zr^4+^ and Ce^4+^ on the Sn site] and aliovalent cations [Al^3+^, Ga^3+^, In^3+^, Sc^3+^, Y^3+^, Gd^3+^ and La^3+^ on the Sn site].

## 2 Computational methods

Classical simulation as established in the GULP (General Utility Lattice Program) code [35] were employed to calculate the defect energetics, construct Li-ion migration pathways together with migration energies and identify favourable dopants on both Li and Sn sites. Long-range Coulombic interactions were used to model the attraction between the oppositively charged ions. Short-range interactions were modelled using repulsion as described by Pauli and attraction as formulated by van der Waals. The short range interactions were described by Buckingham potentials. The Broyden-Fletcher-Goldfarb-Shanno (BFGS) algorithm was employed to perform full geometry optimisation [36]. The forces on the atoms were less than 0.001 eV/Å. The Mott-Littleton method [37] enabled to model point defects and migrating ions. In this method, two regions are defined. The ions in the inner region are relaxed explicitly and ions in the outer region are optimised using approximate quasi-continuum methods. The Li-ion diffusion was calculated considering two nearest neighbour Li vacancies as initial and final configurations. The activation energy of Li ion migration is the local maximum energy along the diffusion path. In the current model, ions have their full charge at dilute limit and defect energies are overestimated. However, it is expected that the trend will be consistent [38]. In this study, isobaric parameters were utilised to calculate formation and migration energies. In previous theoretical work, thermodynamical relations between isobaric parameters and defect energies have been well discussed [39].

## 3 Results and discussion

### 3.1 Crystal structure of Li_8_SnO_6_

**Figure 1:**
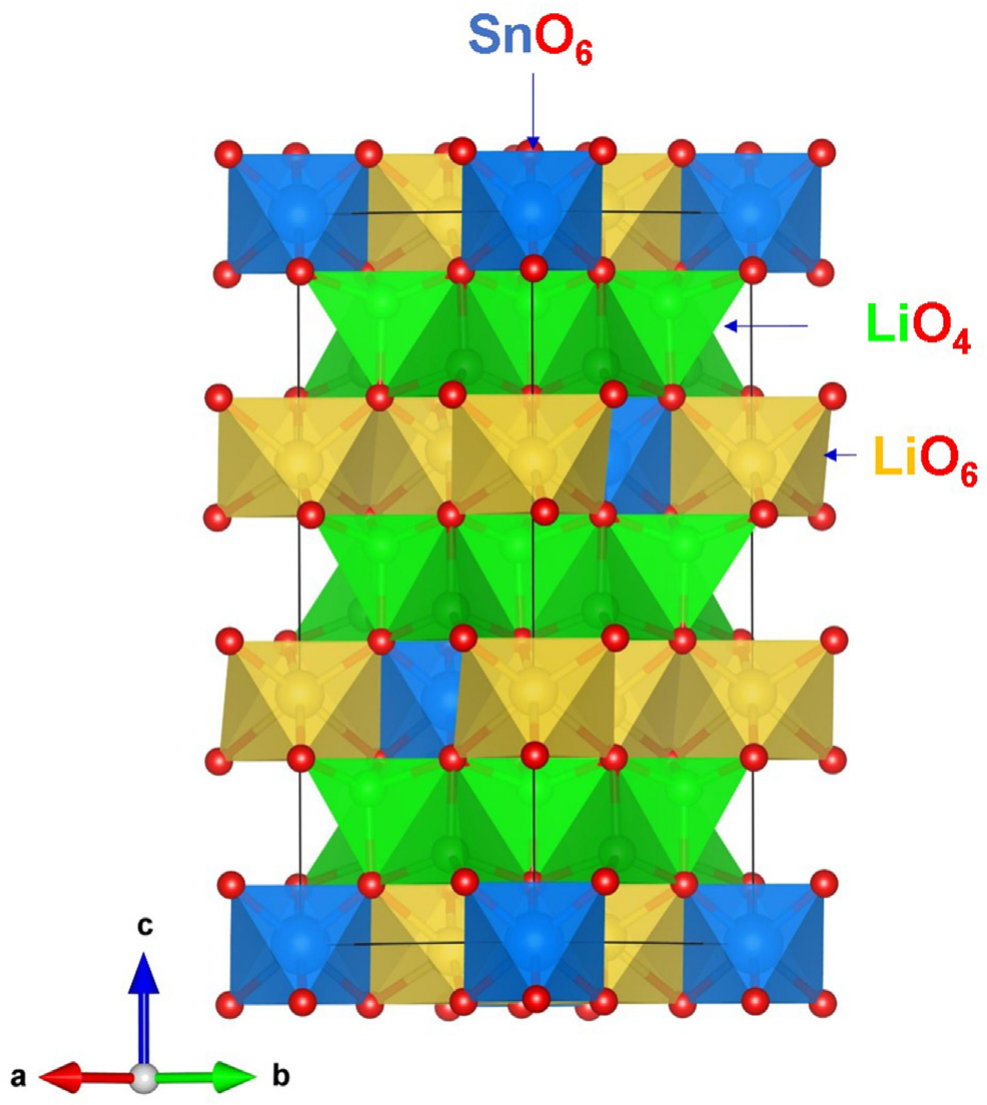
Crystal structure of trigonal Li_8_SnO_6_.

Figure 1 shows the crystal structure of trigonal Li_8_SnO_6_ (space group $R3^{¯}$) [25]. Lattice parameters determined by neutron diffraction refinement are a = b = 5.461 Å, c = 15.278 Å, α = β = 90° and γ = 120° [25]. There are two non-equivalent Li sites present in the lattice. The first Li forms LiO_4_ tetrahedrons whereas the second Li has a coordination of six with adjacent O atoms. The Sn^4+^ ions are coordinated by six O^2‒^ ions forming SnO_6_ octahedrons. Both octahedral and tetrahedral units are interlinked by sharing their corners and edges. To check the quality of the Buckingham potentials (see Table 1) [40, 41, 42], a full geometry optimisation was carried out and calculated lattice parameters were compared with the values reported in the experiment. There is an excellent agreement between the calculated and experimental structural parameters showing the quality of the potentials used in this study (refer to Table 2).

**Table 1:** Buckingham potential parameters used in the classical simulations of Li_8_SnO_6_ [40-42]. Two-body [Φ_*ij*_ (*r*_*ij*_) = *A*_*ij*_ exp (−*r*_*ij*_/*ρ*_*ij*_) — *C*_*ij*_*/r*_*ij*_^6^] where A, ρ and C are parameters reproducing the experimental data. The values of Y and K are shell charges and spring constants respectively.

Interaction	*A*/eV	*ρ*/Å	*C*/eV·Å^6^	Y/e	K/eV·Å^−2^
Li^+^–O^2−^	632.1018	0.2906	0.000	1.000	99999
Sn^4+^ ‒ O^2−^	1414.32	0.3479	13.66	1.000	99999
O^2^–O^2−^	22764.30	0.1490	27.627	‒2.75823	30.211

**Table 2:** Calculated and experimental lattice parameters of trigonal Li_8_SnO_6_.

	Calculated	Experiment [25]	|Δ|(%)
a = b (Å)	5.47	5.46	0.08
c (Å)	15.08	15.28	1.29
α = β (°)	90.00	90.00	0.00
γ (°)	120.00	120.00	0.00
V (Å)^3^	390.14	394.58	1.13

### 3.2 Intrinsic defects

Point defects are important as they can dominate diffusion of ions and alter the behaviour of a material. First, we calculated point defect (vacancies and interstitials) energies and then combined them together with appropriate lattice energies to calculate Schottky and Frenkel defect energies. Anti-site defects were also considered in two different forms (isolated and cluster). In the isolated form cation impurities were considered separately and the same defects were modelled close to each other in the cluster form. The following equations (Eqs. (1), (2), (3), (4), (5), (6), (7), and (8)) describe the defect reactions using Kröger-Vink notation [43].

(1) $$\text{Li Frenkel}:{\text{Li}}_{\text{Li}}^{\text{X}}\to \;V_{\text{Li}}^{\text{′}}+{\text{ Li}}_{\text{i}}^{•}$$

(2) $$\text{Sn Frenkel}:{\text{Sn}}_{\text{Sn}}^{\text{X}}\to V_{\text{Sn}}^{\text{⁗}}+{\text{ Sn}}_{\text{i}}^{••••}$$

(3) $$\text{O Frenkel}:\;{\text{O}}_{\text{O}}^{\text{X}}\to V_{\text{O}}^{••}+{\text{ O}}_{\text{i}}^{\text{″}}$$

(4) $$\text{Schottky}:8{\text{Li}}_{\text{Li }}^{\text{X}}+{\text{ Sn}}_{\text{Sn}}^{\text{X }}+6{\text{ O}}_{\text{O}}^{\text{X}}\to 8{\text{ V}}_{\text{Li}}^{\text{′}}+{\text{ V}}_{\text{Sn}}^{\text{⁗}}+6{\text{ V}}_{\text{O}}^{••}+{\text{ Li}}_{8}{\text{SnO}}_{6}$$

(5) $${\text{Li}}_{2}\text{O Schottky}:\;{\text{Li}}_{\text{Li}}^{\text{X}}+{\text{ O}}_{\text{O}}^{\text{X }}\to 2V_{\text{Li}}^{\text{′}}+V_{\text{O}}^{••}+{\text{ Li}}_{2}\text{O}$$

(6) $${\text{SnO}}_{2}\text{ Schottky}:{\text{Sn}}_{\text{Sn}}^{\text{X}}+2{\text{ O}}_{\text{O}}^{\text{X }}\to V_{\text{Sn}}^{\text{⁗}}+2V_{\text{O}}^{••}+{\text{ SnO}}_{2}$$

(7) $$\text{Li}/\text{Sn antisite }\left(\text{isolated}\right):{\text{Li}}_{\text{Li}}^{\text{X}}+{\text{ Sn}}_{\text{Sn}}^{\text{X }}\to {\text{Li}}_{\text{Sn}}^{\text{‴}}+{\text{Sn}}_{\text{Li}}^{•••}$$

(8) $$\text{Sn}/\text{Li antisite }\left(\text{cluster}\right):{\text{Li}}_{\text{Li}}^{\text{X}}+{\text{ Sn}}_{\text{Sn}}^{\text{X }}\to {\left\{{\text{Li}}_{\text{Sn}}^{\text{‴}}:{\text{Sn}}_{\text{Li}}^{•••}\right\}}^{\text{X}}$$

(9) $$\text{Binding energy }\left(\text{BE}\right):\;E_{cluster}-E_{isolated}$$

Table 3 reports the defect reaction energies. The Li Frenkel (equation 1) is calculated to be the lowest defect energy process with a defect energy of 1.08 eV/defect. Li vacancies needed for the vacancy mediated Li-ion migration will be facilitated by this process. In a previous simulation study [24], the Li-Frenkel was reported to the most favourable defect energy process in Li_2_SnO_3_. The Li–Sn anti-site defect cluster (equation 8) energy is the second most favourable defect with a defect energy of 1.47 eV/defect suggesting that a small percentage of cation mixing (${\text{Li}}_{\text{Sn}}^{\text{‴}}{\text{ and Sn}}_{\text{Li}}^{•••}$) will be present. Anti-site defects have been shown to be important in the ion diffusion of a material [44]. The preference of anti-site defect cluster is due to the exoergic binding of isolated defects (‒3.03 eV) (equation 9). There is no experimental report about Li–Sn anti-site defect in Li_8_SnO_6_ though Li–Sn cation mixing is reported experimentally for Li_2_SnO_3_ [22]. Schottky and Li_2_O Schottky defect energies are calculated to be ~2 eV meaning that Li_8_SnO_6_ may have high ionic diffusion. In particular, Li_2_O Schottky defect energy of 2.08 eV indicates that the loss of Li_2_O is facilitated by the Li-Frenkel. As we discussed earlier, the loss of Li_2_O may degrade the battery performance. However, the O Frenkel energy is 3.40 eV per defect. The loss of Li_2_O can be facilitated further by facilitating the O Frenkel process. High defect reaction energies are noted for the other Schottky and Frenkel defect processes implying that they are not observed at room temperature. Particularly, the Sn Frenkel energy is 5.35 eV/defect showing that this process will only occur at high temperatures. The high defect energy is due to the highly charge defects ($V_{\text{Sn}}^{\text{⁗}}$ and ${\text{Sn}}_{\text{i}}^{••••})$ involved in this process.

**Table 3:** Reaction energies calculated for Schottky, Frenkel and anti-site defects.

Defect process	equation	Reaction energy (eV)	Reaction energy (eV)/defect
Li Frenkel	1	2.16	1.08
Sn Frenkel	2	10.70	5.35
O Frenkel	3	6.80	3.40
Schottky	4	27.98	1.87
Li_2_O Schottky	5	4.16	2.08
SnO_2_ Schottky	6	12.66	4.22
Li/Sn anti-site (isolated)	7	9.00	4.50
Li/Sn anti-site (cluster)	8	2.94	1.47
Binding energy	9	‒3.03	

### 3.3 Diffusion of Li ions

Intrinsic Li-ion migration pathways and their migration energies were next calculated. In general, there is a challenge to determine ion diffusion pathways experimentally. Classical simulation techniques enabled us to construct possible Li-ion diffusion paths and their activation energies. The current simulation technique has been previously used to validate the experimental ion diffusion pathways and predict the possible pathways for materials where it is difficult to determine them experimentally [40, 45, 46, 47].

**Figure 2:**
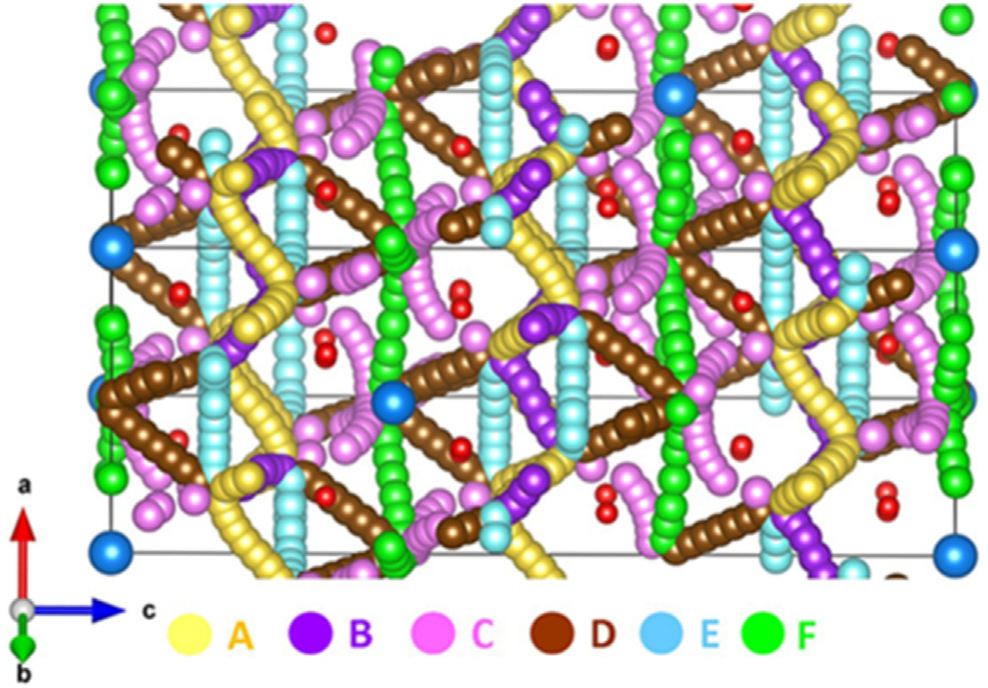
Long range lithium ion diffusion pathways (A–F). Individual Li local hops are represented with different colours.

**Figure 3:**
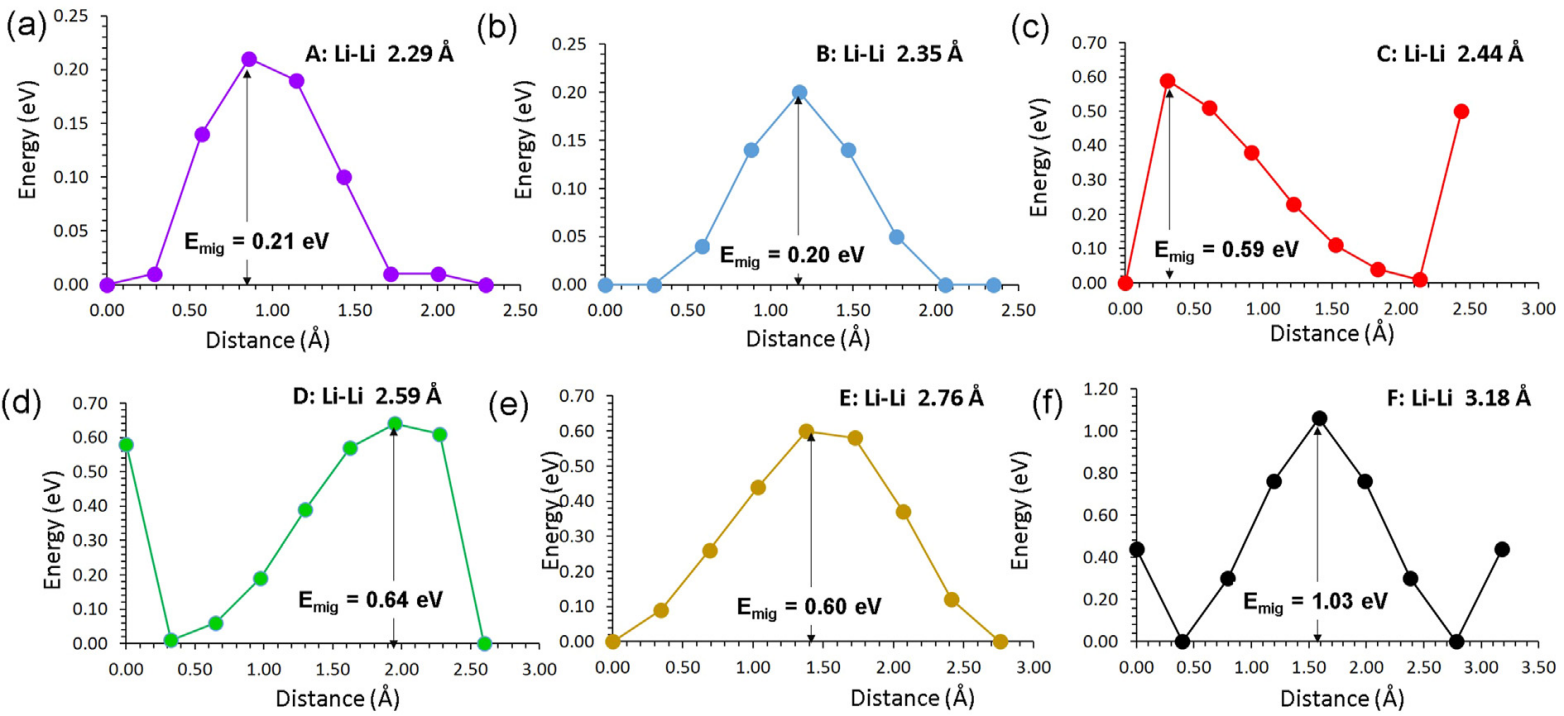
Energy profile diagrams for the local Li hops (A–F) as shown in Figure 2.

Six possible Li local hops (A-F) were identified (refer to Figure 2). Table 4 reports the activation energies calculated for local Li hops together with hop distances. Figure 3 shows the migration barrier for the local Li hops. Local Li hops exhibit a range of activation energies of migration from 0.20 eV to 1.06 eV. In a previous DFT simulation study [27], it has been reported that hopping distances are in the range between 0.43 eV and 1.40 eV. Furthermore, the lowest activation energy of migration is found between two tetrahedral Li sites in agreement with the current simulation though there is an energy difference of 0.23 eV. The difference in the activation energy of migration is due to the calculations employed using different methodologies. In particular, current methodology treated Li as Li^+^ ion during the migration between two adjacent sites. In order to identify long range diffusion paths, local hops were linked in different ways. Five possible pathways were identified. In the first long range pathway (A→B→A→B), Li ion migrates along the *ab* plane in a zig-zag pattern with an overall activation energy of 0.21 eV (refer to Table 5). The second path (B→A→E→A) also exhibits a zig-zag pattern in the *ab* plane with an overall activation energy of 0.60 eV. As local hop D is included in the third and fourth pathways, overall activation energies are slightly high (0.64eV). The fifth pathway consists of only local hops F and exhibits a high activation energy of 1.06 eV. A previous theoretical study predicted that the activation energy for the long range Li-ion migration in Li_2_SnO_3_ is 0.61 eV [24]. The diffusion of Li-ion in Li_8_SnO_6_ is predicted to be faster than that in Li_2_SnO_3_.

**Table 4:** Calculated Li–Li separations and activation energies for the Li-ion migration between two adjacent Li sites (refer to Figure 2). Symbols T_d_ and O_h_ represent Li-ions occupying tetrahedral and octahedral sites.

Migration path	Direction	Li–Li separation (Å)	Activation energy (eV)
A	T_d_↔T_d_	2.29	0.21
B	T_d_↔T_d_	2.35	0.20
C	T_d_↔O_h_	2.44	0.59
D	T_d_↔O_h_	2.59	0.64
E	T_d_↔T_d_	2.76	0.60
F	O_h_↔O_h_	3.18	1.06

**Table 5:** Long range Li ion diffusion paths with corresponding overall activation energies (refer to Figures 2 and 3).

Long range path	Over all activation energy (eV)
A↔B↔A↔B	0.21
B↔A↔E↔A	0.60
B↔A↔D↔D	0.64
A↔A↔D↔C	0.64
F↔F↔F↔F	1.06

### 3.4 Solution of dopants

As material performance can be partly dominated by dopants, we considered a range of monovalent, trivalent and tetravalent cation dopants occupying the Li and Sn sites in Li_8_SnO_6_. The current methodology enabled to test a variety of dopants and identify potential dopants that should be considered experimentally. Aliovalent dopant process needed appropriate charge compensating mechanism. In all cases, appropriate lattice energies were included in the defect reaction equations. Pair-wise potentials used for the dopants are tabulated in the supplementary information (refer to Table 6).

**Table 6:** Interatomic potential parameters used for dopants in the atomistic simulations of Li_8_SnO_6_.

Interaction	*A* (eV)	*ρ* (Å)	*C* (eV·Å^6^)	Y (e)	K (eV·Å^−2^)
Na^+^–O^2−^	1497.830598	0.287483	0.000	1.000	99999
K^+^–O^2−^	1000.3	0.36198	10.569	1.000	99999
Rb^+^–O^2−^	1010.80	0.3793	0.00	1.000	99999
Al^3+^ - O^2−^	1725.20	0.28971	0.000	3.000	99999
Ga^3+^ - O^2−^	2901.12	0.2742	0.000	1.000	99999
Sc^3+^ - O^2−^	1575.85	0.3211	0.000	3.000	99999
In^3+^ - O^2−^	1495.65	0.3327	4.33	3.000	99999
Y^3+^ - O^2−^	1766.40	0.33849	19.43	3.000	99999
Gd^3+^ - O^2−^	1885.75	0.3399	20.34	3.000	99999
La^3+^ - O^2−^	2088.79	0.3460	23.25	3.000	99999
Si^4+^ - O^2−^	283.910	0.320520	10.660	4.000	99999
Ge^4+^ - O^2−^	1497.3996	0.325646	16.00	4.000	99999
Ti^4+^ - O^2−^	5111.7	0.2625	0.00	‒0.10	314.0
Zr^4+^ - O^2−^	985.869	0.3760	0.00	1.35	169.617
Ce^4+^ - O^2−^	1986.83	0.3511	20.40	7.70	291.75

#### 3.4.1 Isovalent dopants

Monovalent and tetravalent dopants were first considered on the Li and Sn sites respectively. The Li site was replaced by monovalent dopants (M = Na, K and Rb) and the reaction process is explained by the following equation (equation 10).

(10) $${\text{M}}_{2}\text{O }+2{\text{ Li}}_{\text{Li}}^{\text{X}}\to 2{\text{M}}_{\text{Li}}^{\text{X}}+{\text{ Li}}_{2}\text{O}$$

**Figure 4:**
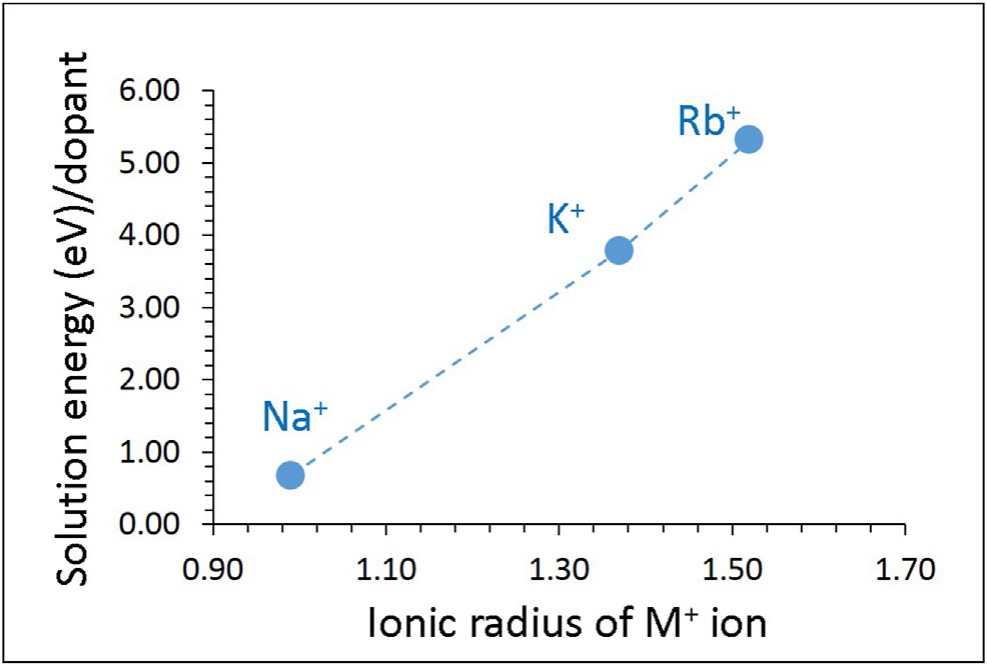
Calculated solution energies of M_2_O (M = Na, K and Rb) with respect to the M^+^ ionic radius in Li_8_SnO_6_.

Solution energies are reported in Figure 4. The promising dopant is the Na with the solution energy of 0.68 eV. The endoergic solution energy is due to the fact that the ionic radius of Na^+^ (0.99 Å) is larger than that of Li^+^ (0.54 Å). Solution energy increases with the increase of ionic radius. There is a significant increase in the solution energy for the K^+^ by ~3 eV compared to that calculated for the Na^+^. The highest solution energy of 5.32 eV is calculated for Rb^+^ implying that doping of Rb on the Li site is not a favourable process.

Tetravalent dopants (M = Si^4+^, Ge^4+^, Ti^4+^, Zr^4+^ and Ce^4+^) were then considered on the Sn site. The following reaction equation describes the doping process (equation 11).

(11) $${\text{MO}}_{2}+{\text{Sn}}_{\text{Sn}}^{\text{X}}\to {\text{M}}_{\text{Sn}}^{\text{X}}+{\text{SnO}}_{2}$$

**Figure 5:**
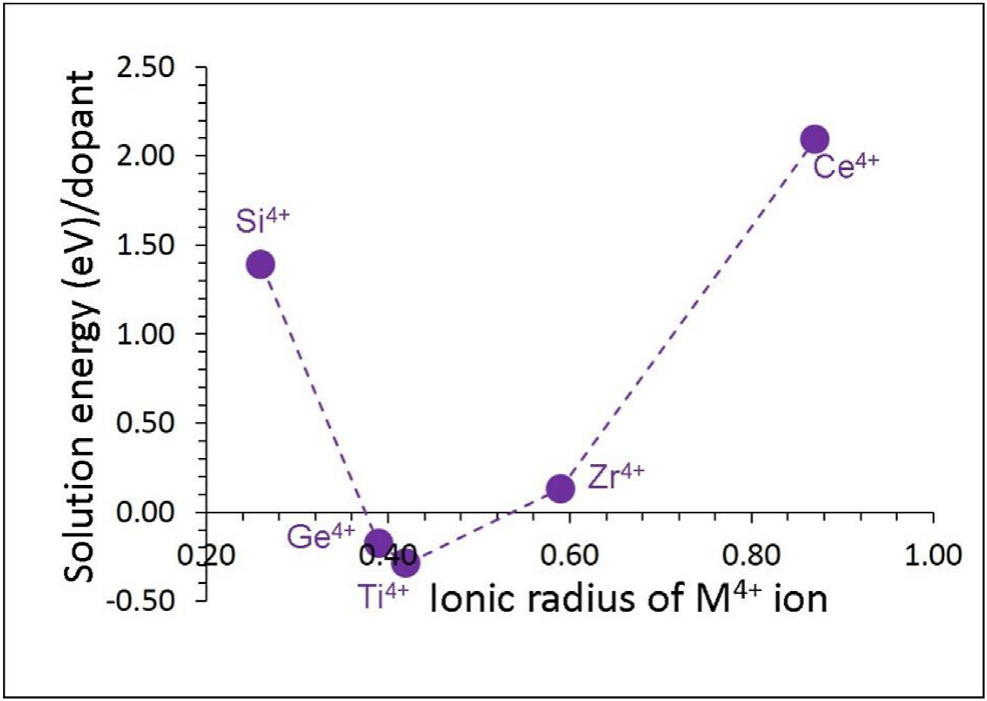
Calculated solution energies of MO_2_ (M = Si, Ge, Ti, Zr and Ce) with respect to the M^4+^ ionic radius in Li_8_SnO_6_.

Exoergic solution energies are calculated for the Ti^4+^ and Ge^4+^, meaning that they are worth trying experimentally. Promising dopant is found to be the Ti with a solution energy of ‒0.29 eV (refer to Figure 5). The solution energy calculated for the Ge^4+^ is higher only by 0.09 eV. The Si exhibits a positive solution energy of 1.39 eV. This can be partly owing to the ionic radius mismatch between Sn^4+^ (0.55 Å) and Si^4+^ (0.26 Å). Doping of Zr^4+^ is endothermic only by 0.13 eV. The Ce^4+^ exhibits the most positive solution enthalpy of 2.09 eV. This is because of the larger ionic radius of Ce^4+^ (0.87 Å) than that of Sn^4+^.

#### 3.4.2 Aliovalent dopants

Trivalent cation dopants including *p*-block, transition and lanthanide elements (M = Al^3+^, Ga^3+^, Sc^3+^, In^3+^, Y^3+^, Gd^3+^ and La^3+^) on the Sn site can introduce lithium interstitials or oxygen vacancies. Additional lithium ions in Li_8_SnO_6_ can increase its capacity. The following defect reaction equation was used to calculate solution energies (equation 12).

(12) $${\text{M}}_{2}{\text{O}}_{3}+2{\text{ Sn}}_{\text{Sn}}^{\text{X}}+{\text{ Li}}_{2}\text{O }\to 2{\text{ M}}_{\text{Sn}}^{•}+2{\text{ Li}}_{\text{i}}^{•}+2{\text{ SnO}}_{2}$$

**Figure 6:**
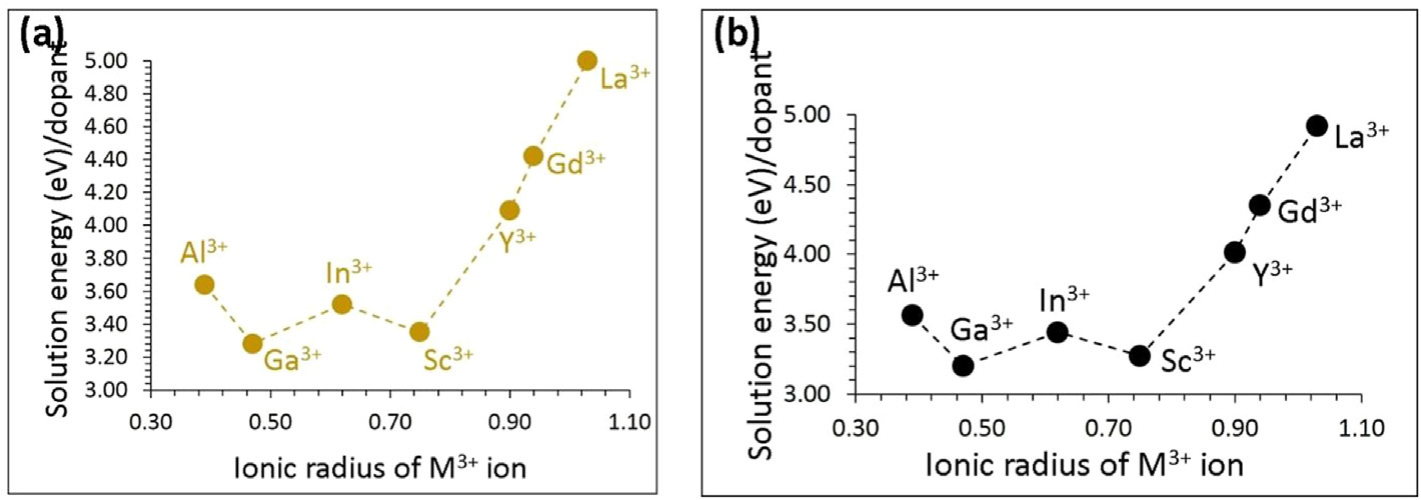
Calculated solution energies of M_2_O_3_ (M = Al, Ga, In, Sc, Y, Gd and La) with respect to the M^3+^ ionic radius introducing (a) Li interstitials and (b) O vacancies in Li_8_SnO_6_.

The lowest solution energy (3.28 eV) is calculated for Ga (refer to Figure 6a). The solution energy calculated for Sc is only higher by 0.07 eV compared that calculated for Ga. Solution energy calculated for Al is 3.64 eV. This is partly due to the ionic radius and charge mismatch between Al^3+^ (0.39 Å) and Sn^4+^ (0.55 Å). A slight increase in the solution energy is observed for Sc^3+^. Solution energies are quite high for other dopants due to their ionic radii deviating from the ionic radius of Li^+^. The largest solution energy is calculated for La^3+^ with a solution energy of 5.00 eV.

The oxygen vacancy formation can be explained by the following reaction equation (equation 13).

(13) $${\text{M}}_{2}{\text{O}}_{3}+2{\text{Sn}}_{\text{Sn}}^{\text{X}}+{\text{Li}}_{2}\text{O }\to 2{\text{ M}}_{\text{Sn}}^{•}+2{\text{V}}_{\text{O}}^{\text{′}}+2{\text{SnO}}_{2}$$

The concentration of oxygen vacancies can facilitate the loss of Li_2_O in this material. The same trend is observed as in the first mechanism (refer to Figure 6b). However, the solution energies are slightly lower than those calculated for the first mechanism indicating that the formation of oxygen vacancies is easier than that of lithium interstitials upon doping of tetravalent cations on the Sn site in Li_8_SnO_6_.

## 4 Conclusions

Classical simulations were employed to examine the intrinsic defect, diffusion and dopant properties of Li_8_SnO_6_. The Li Frenkel is the most favourable intrinsic defect ensuring the formation of Li vacancies needed for the vacancy mediated Li-ion diffusion. The Li-ion migration in this material is fast with a low activation energy of 0.21 eV. Promising isovalent dopants on the Li and Sn sites were Na and Ti respectively. Trivalent dopants were considered on the Sn site to introduce the Li interstitials in order to increase the capacity of this material. Doping with Ga is found to be the efficient strategy for this process. As the same Ga doping process can increase the concentration of oxygen vacancies, it is anticipated that Li_2_O is also favoured by the doping of Ga on the Sn site.

## Declarations

### Author contribution statement

Navaratnarajah Kuganathan: Conceived and designed the study; Performed the experiments; Analyzed and interpreted the data; Wrote the paper.

Andrei L. Solovjov, Ruslan V. Vovk: Conceived and designed the study; Analyzed and interpreted the data.

Alexander Chroneos: Analyzed and interpreted the data; Wrote the paper.

### Funding statement

This research did not receive any specific grant from funding agencies in the public, commercial, or not-for-profit sectors.

### Data availability statement

Data included in article/supplementary material/referenced in article.

### Declaration of interests statement

The authors declare no conflict of interest.

### Additional information

No additional information is available for this paper.
